# Invisible Struggles: Exploring Challenges Faced by Women With Amputation in India

**DOI:** 10.33137/cpoj.v7i1.44002

**Published:** 2024-10-14

**Authors:** J Alam, A Joshi, N Mir, N Chawla, S Sagar

**Affiliations:** 1 Division of Trauma Surgery and Critical Care, Jai Prakash Narayan Apex Trauma Centre, All India Institute of Medical Sciences, New Delhi, India.; 2 Department of Psychiatry, All India Institute of Medical Sciences, New Delhi, India.

**Keywords:** Amputation, Rehabilitation, India, Women with Amputation, Prosthesis, Prosthetics, Quality of Life, Satisfaction, Disability

## Abstract

Women in India, particularly those with amputation, face significant challenges, including but not limited to, unequal prosthetic access and satisfaction, societal discrimination, and the physical and emotional consequences of amputation. These challenges are further exacerbated by gender biases towards access to education and socioeconomic factors, which increases their vulnerability to unemployment and mental health issues. This article emphasizes the urgent need for affordable and customizable prosthetic options tailored to the unique needs of women with amputation, particularly those from low-income backgrounds who often face neglect. Thus, addressing these disparities would significantly enhance their overall well-being and independence.

The 2019 Global Burden of Diseases (GBD) report highlighted that India sees about 23,500 new cases of people with amputation each year, with men making up the majority—around 20,200—while approximately 3,300 are women.^1^ Older women, especially those over 70, are at a heightened risk for traumatic amputations due to osteoporotic changes, according to GBD data.^2^ However, in our population, Road Traffic Injuries (RTIs) emerge as the leading cause of amputations, followed by train-associated injuries, attributed to lax laws and an increasing population.^3^ Additionally, gender plays a role in prosthetic access and fitting, as fewer women (42.9%) successfully receive prosthetic limbs upon discharge compared to men (68.6%).^4^ This disparity may stem from concerns related to the appearance of the prosthesis and its bulkiness.

In patriarchal societies like India, there is often a tendency to prioritize men's comfort over women's needs, which can result in the dismissal of women's genuine concerns as “emotional” or “natural phenomena”.^5^ This societal bias may contribute to higher prosthesis rejection rates among women as compared to men. Additionally, women with disabilities face a dual form of discrimination—one based on gender and the other on their physical disability. This discrimination, combined with lower literacy rates, significantly reduces their job opportunities.^6^ The lack of empathy in Indian society towards individuals with disabilities is not a secret, especially if they are women, it creates a sense of insecurity in them about their future. They often fear abandonment by their husband or in-laws or have negative views on marriage prospects. In some instances, parents of unmarried daughters do not consent for amputation fearing their uncertain future. Consequently, women with amputations are seen as a “burden,” making them more vulnerable than both able-bodied women and disabled men.

Disability can either cause or result from poverty, creating a vicious cycle that is further exacerbated by stigma and the denial of basic rights and opportunities, particularly for women.^1^ In India, only a handful of organizations such as those supported by the Ministry of Social Justice and Empowerment and Ministry of Women and Child Development under the Government of India offers prosthetic devices at little or no cost to support individuals with disabilities. However, these prosthetic devices are often bulky, outdated in technology, and not well-suited to women needs.^7^ Apart from this, factors such as frequent volumetric changes in the residual limb, patient’s age, comorbidities, and the level of amputation often contribute to the rejection of prosthetic devices. Moreover, the side of the amputation can also play a role in this rejection, especially if it involves the dominant side, which is more relevant in case of upper extremity amputation. This can lead to a diminished sense of body perception, which significantly impacts performance, making individuals slower and more self-conscious while limiting their overall function.^8^

Moreover, in India, women from low socioeconomic backgrounds frequently engage in physically demanding jobs, such as domestic work, agriculture, and manual labour, which necessitate floor sitting, mobility, and stamina. Unfortunately, the affordable prosthetic options available to these women often fall short in providing the functionality required for these strenuous tasks. This inadequacy can lead to feelings of frustration, disappointment, and various health issues.

The literature on people with amputation revealed that they often experience anxiety, depression, and post-traumatic stress disorder. However, women are particularly more vulnerable to depression following an amputation than men. If the amputation involves the upper limb, this risk doubles for women, as the upper limb is crucial for self-expression, self-care, and communication, leading to a greater functional loss compared to lower limb amputations. Moreover, if depression is diagnosed, the likelihood of unemployment increases tenfold. Even though men are more frequently subjected to amputations, women generally experience worse outcomes.^9–11^

To address the specific challenges that women face in India, it is essential to provide affordable yet customizable prosthetic solutions that can support their unique physically demanding work schedules. The low-cost customizable prosthetic devices need to be less bulky and comparatively less in weight for improved functionality. A recent innovation by a prestigious Indian Institute offers a high-end prosthetic feature of cross-legged sitting in a low-cost above-knee prosthesis, holds promise in addressing this gap by not only making them independent but also allowing them to perform long periods of sitting, standing, or squatting, that are typically seen as a royalty for a non-affording person with amputation.^12^

Furthermore, adopting a user-centered design approach that takes into account the experiences of women with amputations during the customization process can highlight essential details that might otherwise be missed. A more secure and snug-fitting suspension that minimizes bulkiness would also be particularly attractive from a cosmetic perspective for women. Additionally, addressing specific needs related to menstrual hygiene or pregnancy is vital, especially for those with higher levels of lower limb amputation where the proximal brim of the socket is a problem. Consequently, providing well-fitted and functional prosthetics is the way of empowering women, enhancing their physical and mental well-being, and reducing disparities in access to prosthetic use. This enables them to lead active, dignified lives, irrespective of their economic status or disability.

## DECLARATION OF CONFLICTING INTERESTS

The authors have no conflicts of interest to disclose. The manuscript has been read and approved by all the authors.

## SOURCES OF SUPPORT

None.
