# Corrigendum: Invisible Struggles: Exploring Challenges Faced by Women with Amputation in India

**DOI:** 10.33137/cpoj.v8i2.46384

**Published:** 2025-12-31

In the article mentioned above^1^, published in Volume 7, Issue 1, 2024, the authors cited Mishra et al. (2020)^2^ for the following statement:

“The 2019 Global Burden of Diseases (GBD) report highlighted that India sees about 23,500 new cases of people with amputation each year, with men making up the majority—around 20,200—while approximately 3,300 are women.”

These statistics were incorrectly attributed to the 2019 Global Burden of Diseases (GBD) report and were originally reported in Mohan D (1986).^3^ These figures do not represent current national statistics.
